# Evaluation of gastroprotectant administration in hospitalized cats in a tertiary referral hospital

**DOI:** 10.1177/1098612X231201769

**Published:** 2023-10-24

**Authors:** Tarini V Ullal, Stanley L Marks, Janny V Evenhuis, Monica E Figueroa, Leah K Pomerantz, Lauren R Forsythe

**Affiliations:** 1 Department of Medicine and Epidemiology, University of California-Davis School of Veterinary Medicine, Davis, CA, USA; 2 VCA East Bay Veterinary Emergency Hospital, Antioch, CA, USA; 3 Tufts Cummings School of Veterinary Medicine, North Grafton, MA, USA; 4 Department of Veterinary Clinical Medicine, University of Illinois College of Veterinary Medicine, Urbana, IL, USA

**Keywords:** Acid suppressant, proton pump inhibitor, histamine-2 receptor antagonist, omeprazole, pantoprazole, famotidine, sucralfate, gastrointestinal ulcer, erosion

## Abstract

**Objectives:**

The primary objective of this study was to evaluate the prescription patterns and appropriateness of the use of gastroprotectant medication in cats.

**Methods:**

Pharmacy dispensation logs from an academic tertiary referral center were reviewed between 1 January 2018 and 31 December 2018. Cats that were administered proton pump inhibitors (PPIs), histamine-2 receptor antagonists (H2RAs), sucralfate, misoprostol, antacids or a combination were included. Data regarding medication, dosage, formulation, duration of administration, completeness of discharge instructions and clinical rationales for administration were obtained from medical records. The appropriateness of gastroprotectant use was assessed according to the American College of Veterinary Internal Medicine consensus statement guidelines.

**Results:**

Of the 110 cases, 67 (60.9%) were prescribed a gastroprotectant medication without an appropriate indication. The most common reason for prescription was acute kidney injury in 26/67 (38.8%). PPIs were the most common gastroprotectant medication administered in 95/110 (86.3%) cats, followed by sucralfate in 18/110 (16.4%) and H2RAs in 11/110 (10%). Of the 35 cases in which gastroprotectant therapy was indicated, the medication chosen or dosage administered was considered suboptimal in 16 (45.7%). Instructions regarding the duration of administration, potential adverse effects and timing of administration in relation to meals or other medications were inconsistently provided in discharge instructions to pet owners. Of the 29 cases discharged with omeprazole, only 13 (44.8%) instructions included a duration of administration, while 6 (20.7%) recommended continuing gastroprotectants indefinitely until further notice, 16 (55.2%) discussed the timing of the administration in relation to a meal and six (20.7%) mentioned potential adverse effects; none advised tapering of omeprazole before discontinuation.

**Conclusions and relevance:**

When prescribed, gastroprotectant medications were frequently prescribed injudiciously to cats in this referral population over a 12-month period. Discharge instructions to pet owners also often lacked information and recommendations regarding optimal administration, potential adverse effects, and tapering or discontinuation of the medications.

## Introduction

Gastroprotectant medications, particularly acid suppressants such as proton pump inhibitors (PPIs) and histamine-2 receptor antagonists (H2RAs), are widely prescribed in human and small animal veterinary medicine to prevent and treat acid-related esophageal and gastrointestinal (GI) mucosal injury.^1–10^ Frequent use in humans and animals has led to growing concerns of overutilization and inappropriate prescriptions of gastroprotectants.^1,7–14^ In humans, excessive acid suppressant prescription^1,14–16^ has resulted in an excess expenditure on PPIs of nearly $10 billion per year in the USA^17^ and increased recognition of adverse effects associated with chronic, prolonged use of PPIs, such as micronutrient deficiencies, small intestinal bacterial overgrowth, pneumonia, osteoporosis, dementia and chronic renal insufficiency.^18,19^ Excessive PPI use has been observed in small animal veterinary medicine as well, mainly in dogs.^7,8,10^ Side effects, such as anorexia, vomiting, diarrhea,^20^ intestinal dysbiosis,^21,22^ exacerbation of non-steroidal anti-inflammatory (NSAID) medication injury^23^ and rebound hypersecretion after abrupt discontinuation,^22,24^ have been documented or postulated.

Concerns regarding the overuse of gastroprotectants in veterinary medicine led to the publication of an American College of Veterinary Internal Medicine (ACVIM) consensus statement^25^ that included a set of guidelines on the rational administration of gastroprotectant medications to dogs and cats. The primary conclusions were that gastroduodenal ulceration or erosion (GUE), esophagitis and exercise-induced GUE were strong indications for gastroprotectant therapy, and that PPIs achieved superior acid suppression compared with H2RAs. Recommendations for dosage and tapering of gastroprotectants after chronic use were also included. Despite heightened awareness and advocacy to reduce gastroprotectant overuse in human and veterinary medicine,^19,25,26^ PPIs are commonly prescribed to dogs without justified indications^7,8,10^ and at incorrect dosage regimens.^7,8^ However, whether the same trends occur in feline medicine is unknown; therefore, evaluating the use of PPIs and other gastroprotectants in cats is warranted.

The primary objective of this retrospective study was to document and evaluate the patterns of gastroprotectant prescription and appropriateness of use in a cohort of cats that presented to an academic tertiary referral hospital during a 12-month period. We hypothesized that gastroprotectants would frequently be prescribed and administered injudiciously to cats with suboptimal recommendations pertaining to dosage, adverse effects and tapering of the medications in the discharge instructions.

## Materials and methods

### Case selection criteria

An electronic search of the pharmacy prescription dispensing log from the University of California, Davis, Veterinary Medical Teaching Hospital was retrospectively performed by three investigators (MEF, JVE and LKP) to identify cats prescribed gastroprotectant medications (PPIs, H2RAs, sucralfate, misoprostol, antacids such as aluminum hydroxide, magnesium hydroxide, calcium carbonate or a combination) over a 12-month period (1 January 2018 to 31 December 2018). Cases were included based upon species (cats), pharmacy dispensation of the gastroprotectant and medical record documentation of its administration. Gastroprotectants could be administered in hospital, discharged to go home or both. For cats in which administration of the relevant medications spanned several visits for the same reason, one single prescription event was logged based upon the first visit in the study period. Cases with inadequate information in the medical record to confirm that gastroprotectants had been administered in hospital or prescribed were excluded.

### Review of medical records

The following information was obtained from medical records of cats that met the inclusion criteria:

1. Signalment, including age, sex and breed
2. Body weight (kg)
3. Whether gastroprotectants were administered within 48 h before presentation to the University of California, Davis, Veterinary Medical Teaching Hospital (data collected from medical history or referral veterinarian paperwork)
4. Clinical signs documented in the medical record at the time of prescribing gastroprotectants
5. Chronicity of the noted clinical signs
6. Rationale for prescribing gastroprotectants
7. Concurrently administered medications
8. Gastroprotectants prescribed in hospital including route of administration, form, dosage and duration
9. Gastroprotectants sent home and directions for administration provided to owner, including route of administration, form, dosage, duration and administration in relation to meals and other medications
10. If a PPI was prescribed to go home, directions for tapering

Two board-certified internists with advanced expertise in gastroenterology independently reviewed the medical records from 20/110 cases to identify the rationales for prescribing gastroprotectants. One or more rationales for prescription was selected from a list developed by the two internists (Table 1), guided by the ACVIM 2018 consensus statement. If a justification for gastroprotectant use was not apparent from the medical record, this was noted. After identifying a rationale for prescription, guidelines published in the ACVIM 2018 consensus statement were used to classify whether gastroprotection was (1) appropriate, (2) equivocal, (3) inappropriate or (4) indeterminate owing to insufficient information. After reviewing the first 20 cases independently, the two internists reached a consensus on the categorization for the first 20 cases and developed a categorization scheme (Table 2), which was then applied to all cases.

**Table 1:** List of rationales for prescribing gastroprotectant therapy

1. AKI without diagnosed GUE
2. CKD without diagnosed GUE
3. Azotemia (could not identify renal or pre-renal, or acute vs chronic) without diagnosed GUE
4. Anemia due to possible GI bleeding
5. Clinical signs of GI bleeding (hematemesis, melena, hematochezia or a combination of two or more)
6. Coagulopathy (disorders of primary, secondary or tertiary hemostasis)
7. Discordant blood urea nitrogen:creatinine ratio due to possible GI bleeding (>30:1)^27^
8. Esophagitis – suspected based on clinical signs of gagging, retching, hard swallowing, regurgitation, ptyalism, odynophagia or some combination thereof or confirmed based on esophagoscopy findings
9. Gastroesophageal reflux disease – confirmed based on diagnostic imaging, such as swallowing fluoroscopy
10. Non-specific GI clinical signs of unknown etiology, presumed gastritis or gastroenteritis
11. Suspected IBD or intestinal small cell lymphoma (based on history, clinical signs, laboratory findings and ultrasonographic imaging of thickened intestinal wall layers) or histologically confirmed
12. Diagnosed GUE with ultrasound, endoscopy or intraoperatively
13. Pancreatitis
14. Intra-abdominal or GI surgery
15. Hepatic disease (specific hepatic disease was recorded)
16. Toxin ingestion (specific toxin was recorded)
17. NSAID administration
18. Corticosteroid use
19. Mast cell neoplasia
20. Oral lesions such as stomatitis or ulcerations

AKI = acute kidney injury; CKD = chronic kidney disease; GI = gastrointestinal; GUE = gastroduodenal erosion or ulceration; IBD = inflammatory bowel disease; NSAID = non-steroidal anti-inflammatory drug

**Table 2:** Categorization scheme used in assessing the appropriateness of gastroprotectant administration

1a = Gastroprotectants were indicated and appropriate for the following indications:
1 Clinical, biochemical, ultrasonographic, endoscopic or surgical evidence of GUE
a. Clinical: hematemesis, melena of GI origin (not swallowed epistaxis) or both
b. Hematological:
i. Macrocytic, hypochromic, regenerative anemia or microcytic, moderate to severe anemia
c. Biochemical: Discordant blood urea nitrogen:creatinine ratio along with one other piece of evidence from item 1 (1a, 1b, 1d or 1e) or renal and pre-renal causes ruled out
d. Ultrasonographic: GI ulcer, gas tracking through the GI wall
e. Endoscopic or surgical: erosions or ulceration visualized endoscopically or intraoperatively
2 Esophagitis
a. Clinical signs of esophagitis: gagging, retching, hard swallowing, salivation, odynophagia or a combination
b. Endoscopic evidence of esophagitis
c. Regurgitation, especially if
i. Dysmotility on fluoroscopic swallow study
ii. Megaesophagus
iii. Occurred under or after anesthesia
iv. Recent doxycycline, clindamycin, NSAID administration or anesthesia
3 Gastroesophageal reflux with or without a hiatal hernia
a. Identified on fluoroscopic swallow study
4 Mast cell neoplasia, gastrinoma
5 NSAID toxicity, overdose or NSAID + corticosteroids co-administration or consecutive administration
a. Misoprostol was justified in cases of aspirin toxicity only^28^ and not other NSAID or corticosteroid-induced GUE
6 Sucralfate for lingual or oral ulceration from calicivirus, stomatitis or renal disease^29,30^
1b = Gastroprotectant therapy was indicated; however, the medication, dose or frequency was suboptimal for the following indications:
1 H2RA, sucralfate, misoprostol or antacid chosen over PPI
2 PPI or H2RA administered q24h instead of q12h
3 PPI or H2RA dose <0.7 mg/kg without mentioned justification (such as renal disease or dosing based on lean body weight if patient was obese) for dose reduction
4 Misoprostol for non-aspirin NSAID toxicity
2 = Gastroprotectant therapy was deemed equivocal for the following indications:
1 Severe persistent vomiting defined as several daily episodes for the past 2 or more consecutive days^31^ with no evidence of esophagitis or GUE
2 IRIS CKD stage 4 with no evidence of GUE
3 Gastric, intestinal (excluding small cell lymphoma), pancreatic neoplasia with no evidence of GUE
4 Microcytosis or discordant blood urea nitrogen:creatinine ratio, but mild anemia with concomitant renal disease
5 Thrombocytopenia or coagulopathy-induced GI bleeding
6 Pre- or post-coil embolization for intrahepatic portosystemic shunts
3 = Gastroprotectant therapy was not indicated for the following diseases or scenarios in the absence of evidence for GUE (see item 1a, 1 for evidence of GUE) or esophagitis (see item 1a, 2 for evidence of esophagitis):
1 Renal disease – cats with AKI or chronic IRIS CKD stage 1, 2 or 3
2 Pancreatitis
3 Foreign body
4 Dietary indiscretion
5 GI surgery
6 Inflammatory bowel disease or small cell lymphoma
7 Non-specific GI signs of inappetence, vomiting, weight loss, diarrhea of unknown etiology
8 Cholangiohepatitis or hepatic lipidosis^32–34^
4 = Could not determine whether gastroprotectant therapy was warranted due to insufficient information in the medical record

AKI = acute kidney injury; CKD = chronic kidney disease; GI = gastrointestinal; GUE = gastroduodenal erosion or ulceration; H2RA = histamine-2 receptor antagonist; IRIS = International Renal Interest Society; NSAID = non-steroidal anti-inflammatory drug; PPI = proton pump inhibitor

## Results

### Description of the cohort

Of 5649 cats seen at the University of California, Davis, Veterinary Medical Teaching Hospital during the 12-month evaluation period, a total of 110 (2%) cats met the inclusion criteria. The most common breeds were domestic shorthair (62/110, 56.4%), domestic longhair (23/110, 20.9%), domestic mediumhair (7/110, 6.4%), Siamese (4/110, 3.6%) and Maine Coon (3/110, 2.7%). Sex distribution was 2/110 (1.8%) intact males, 2/110 (1.8%) intact females, 55/110 (50.0%) castrated males and 51/110 (46.4%) spayed females. The median age and weight were 10.3 years (interquartile range [IQR] 5.5–14 years) and 4.4 kg (IQR 4.2–5.7 kg), respectively.

In the 48 h before presentation to the University of California, Davis, Veterinary Medical Teaching Hospital, 20/110 (18.2%) cats had already received one or more doses of gastroprotectant medications administered by the pet owner or the referring veterinary clinic. The most common medication was famotidine in 12/20 (60%) cats. The most common clinical signs reported on presentation were vomiting (49/110, 44.5%), inappetence (26/110, 23.6%) and anorexia (21/110, 19.1%). Almost half (42/88, 47.7%) the cats with available information regarding duration of clinical signs presented with signs of <3 days, 30/88 (34.1%) presented with signs of >3 days but <3 weeks and 16/88 (18.2%) cats presented with signs >3 weeks.

After presentation to the University of California, Davis, Veterinary Medical Teaching Hospital, 95/110 (86.4%) cats were administered a PPI, 11/110 (10.0%) were administered an H2RA and 18/110 (16.4%) were administered sucralfate. The specific medication selected and medians with IQRs for dose, frequency and duration of administration for each gastroprotectant are provided in Table 3. No cats received misoprostol or antacid medications. Concurrent medications were administered in 97/110 (88.2%) cases, of which the most common were maropitant (62/97, 63.9%), buprenorphine (33/97, 34.0%) and amoxicillin-clavulanic acid, amoxicillin, ampicillin or ampicillin-sulbactam (31/97, 32.0%).

**Table 3:** Summary of gastroprotectant medications, dose, frequency, duration and route of administration in 110 cats in a tertiary referral hospital

	Number of cats	Dose (mg/kg or g*)	Frequency	Duration of administration (days)	Route of administration
Pantoprazole	77/110 (70)	0.97 (0.9–1)	q12h: 62/72 (86.1) q24h: 8/72 (11.1) q12–24h: 1/72 (1.4) Once: 1/72 (1.4)	2 (1–4)	IV (77/77)
Omeprazole	29/110 (26.4)	1.1 (0.8–1.4)	q12h: 17/29 (58.6) q24h: 12/29 (41.4)	8 (6–14)	PO (29/29)
Famotidine	11/110 (10.0)	0.6 (0.43–0.84)	q12h: 6/9 (66.7) q24h: 3/9 (33.3)	3 (3–10)	PO (10/11) IV (1/11)
Sucralfate	18/110 (16.4)	0.25 (0.25–0.5)*	q8h: 11/18 (61.1) q12h: 3/18 (16.7)	8.5 (3.4–13.8)	PO (18/18)

Data are n (%) or median (IQR)

IQR – interquartile range; IV = intravenous; PO = per os

* The dose in grams only pertains to the drug, Sucralfate, and not to any of the other drugs

### Indications and appropriateness of gastroprotectant prescription

Most cases (71/110, 64.5%) were prescribed gastroprotectants for two or more reasons. The most common rationales for prescription were AKI (29/110, 26.4%), inflammatory bowel disease (IBD) or GI small cell lymphoma (16/110, 14.5%), GI signs of unknown etiology and presumed gastroenteritis (14/110, 12.7%), pancreatitis (12/110, 10.9%) and CKD (11/110, 10.0%). None of the 11 cats prescribed gastroprotectant therapy for CKD had IRIS stage 4 disease.

A prescription of gastroprotectants was classified as 1 (appropriate) in 35/110 (31.8%) cats, as 2 (equivocal) in 7/110 (6.4%) cats, as 3 (inappropriate) in 67/110 (60.9%) cats and as 4 (indeterminate) owing to insufficient information in 1/110 (0.9%) cases. Of the 35 cases in which gastroprotectant therapy was appropriate, a gastroprotectant prescription was classified as 1b (suboptimal) in 16 (45.7%) cats (see Table 1 in the supplementary material). In cases where gastroprotectant therapy was deemed inappropriate, the most common rationales lacked evidence of GUE. These included AKI in 26/67 (38.8%) cats, IBD or small cell GI lymphoma in 9/67 (13.4%) cats and GI signs of unknown etiology in 8/67 (11.9%) cats. Of the 35 cases in which gastroprotectant administration was deemed appropriate, the most common rationales were evidence of GUE (20/35, 57.1%) or esophagitis (11/35, 31.4%).

### Proton pump inhibitors

Of the 110 cats, 77 (70.0%) were prescribed PPIs in hospital at the University of California, Davis, Veterinary Medical Teaching Hospital. All 77 cats received pantoprazole intravenously, of which four (5.2%) were subsequently transitioned to oral omeprazole in hospital and 11 (14.3%) were discharged with oral omeprazole. The dose of pantoprazole and frequency of administration in hospital were available for 72/77 (93.5%) cats. The median dose was 0.97 mg/kg IV (IQR 0.9–1.0 mg/kg) and the majority (62/72, 86.1%) of feline patients in hospital were administered pantoprazole q12h. A smaller number of cats (8/72, 11.1%) were administered pantoprazole q24h. One cat was administered pantoprazole q12h then tapered down to q24h. Another cat was only administered pantoprazole once during hospitalization. The median duration of pantoprazole administration in hospital was 2 days (IQR 1–4 days). Of the 77 cats prescribed PPIs, six (7.8%) received concomitant H2RAs and 11 (14.3%) received concomitant sucralfate.

Of the 110 cats, 29 (26.4%) were prescribed a PPI at discharge; in all cases, the PPI was omeprazole. Omeprazole was dispensed as a tablet (26/29, 89.7%) or as an oral suspension (3/29, 10.3%). Discharge instructions included a dose and frequency of administration in 28/29 (96.6%) cases and a duration of administration in 13/29 (44.8%) cases. Discharge instructions included information on administration in relation to a meal in 16/29 (55.2%) cases and potential adverse effects in 6/29 (20.7%) cases. None of the discharge instructions discussed administration of omeprazole with other medications, nor tapering of omeprazole before discontinuation. The median dose of omeprazole prescribed at discharge was 1.1 mg/kg (IQR 0.8–1.4 mg/kg). The majority of cats (17/29, 58.6%) were prescribed omeprazole q12h and the rest were prescribed q24h (12/29, 41.4%). The median prescribed duration of administration was 8 days (IQR 6–14 days), but in 6/29 (20.7%) cases, the discharge instructions advised to administer the medication indefinitely or until otherwise directed.

### Histamine-2 receptor antagonists

Of the 110 cats, 11 (10.0%) were prescribed an H2RA, which was famotidine in all cases. Most cats (9/11, 81.8%) were prescribed famotidine at discharge and the others in hospital (2/11, 18.2%). Famotidine was prescribed in tablet form in all nine cases at discharge. For the two hospitalized cases, famotidine was administered intravenously in one and orally in tablet form in the other.

The discharge instructions to owners of those cats discharged with famotidine revealed that 3/9 (33.3%) included a dose, 3/9 (33.3%) included a frequency of administration and 6/9 (66.7%) included a recommendation to administer the medication for a finite duration (n = 2) or indefinitely (n = 4). Among the nine cases, seven (77.8%) discharge instructions directed owners to administer the medication as previously prescribed. One of nine cases (11.1%) included information on administration relative to meals and recommended administration 30 mins before a meal. None included information on administration relative to other medications, nor discussed potential adverse effects. Of all 11 cats administered famotidine, dose, frequency and duration of administration were available for 9/11 (81.8%) cats. The median dose of famotidine was 0.60 mg/kg (IQR 0.43–0.84 mg/kg). Most cats (6/9, 66.7%) were prescribed H2RAs q12h. Fewer (3/9, 33.3%) were prescribed q24h. The median duration of administration was 3 days (IQR 3–10 days).

### Sucralfate

Of the 110 cats, 18 (16.4%) were prescribed sucralfate, of which 15/18 (83.3%) cats were prescribed the drug to go home and 7/18 (38.9%) were prescribed it in hospital. Sucralfate was administered orally as a tablet dissolved in water in 9/18 (50.0%) cats and as an oral suspension in 7/18 (38.9%). In one additional instance, it was prescribed for administration as a partial tablet, and in a separate instance, it was unknown whether the tablet was dissolved.

The discharge instructions specified dose and frequency of administration in 14/18 (77.8%) cats and duration of administration in 7/18 (38.9%) cats. Among 18 instructions, 12 (66.7%) included recommendations for administration relative to meals and advised giving on an empty stomach (n = 2), 30 mins before food (n = 1), or either 60 mins before or 120 mins after food (n = 9). The administration of sucralfate was recommended at least 1–2 h apart from other medications in 3/18 (16.7%) cats. No discharge instructions discussed potential adverse effects. The median dose of sucralfate was 0.25 g (IQR 0.25–0.5 g). The median duration of administration was 8.5 days (IQR 3.4–13.8 days). Most of the cases (11/18, 61.1%) were prescribed sucralfate q8h and 3/18 (16.7%) cases were recommended q12h.

## Discussion

The present study sought to determine the severity and frequency of misuse of gastroprotectant medications in cats that were presented to a tertiary referral veterinary hospital and prescribed one or more gastroprotectants during a 12-month period. The key findings were that 61% of cats were prescribed gastroprotectants for indications unsupported by the ACVIM consensus statement. In 50% of the cases in which gastroprotectants were prescribed for approved indications, the medication, dose or frequency of administration was suboptimal for the management of acid-related disorders. Furthermore, none of the discharge instructions were comprehensive in providing all the necessary information regarding the timing of administration relative to a meal or other medications, duration of administration, tapering and potential adverse effects.

Of the 110 cats in this study, gastroprotectants were prescribed for indications approved by the consensus statement in only 32% of cases.^25^ For the cases in which gastroprotectant therapy was not indicated, the five most common reasons for prescription included AKI, feline chronic enteropathy, gastroenteritis, pancreatitis and CKD. Thus, when gastroprotectants were inappropriately prescribed, they were most often prescribed to treat cats with renal or GI disease without confirmatory evidence of GUE.

Although the ACVIM consensus statement discourages gastroprotectant use in cats with IRIS stages 1–3 CKD in the absence of GUE, its use in cats diagnosed with AKI or chronic enteropathy is not discussed, perhaps because the literature documenting GUE in affected cats is minimal.^35,36^ The consensus statement mentions that acid suppression might be indicated in cats with IRIS stage 4 CKD^25^ based on evidence in humans.^37,38^ Acute renal failure is one of the independent risk factors for GI bleeding in human patients^39^ and acute GI bleeding is a potential, although uncommon, complication of IBD in humans.^40^ Thus, gastroprotection might be warranted in some cases of renal disease or chronic enteropathy to treat or prevent GUE,^41–43^ but guidelines in human medicine only recommend ulcer prophylaxis if there are additional risk factors such as mechanical ventilation or sepsis.^44^ Furthermore, the prevalence of GUE in cats is thought to be low,^35,36^ and the risk of adverse effects could outweigh the benefits. Only 5% of cats undergoing gastroduodenoscopy over a 4-year period were diagnosed with GUE^45^ and omeprazole is known to cause intestinal dysbiosis,^22^ which could possibly accelerate the severity or progression of GI disease. Cats with CKD do not typically have gastric hyperacidity, hypergastrinemia^43^ or gastric ulceration^46^ to warrant acid suppression, and the administration of PPIs in humans can trigger AKI and accelerate progression to end-stage renal disease by causing acute interstitial nephritis,^47^ hypomagnesemia,^48^ and imbalances in calcium and phosphorous.^49^ Although there is no definitive evidence that these adverse renal outcomes occur with the use of PPIs in cats with CKD^50^ or AKI, they should be considered as plausible sequelae and further studies are required. Thus, the authors considered prophylactic gastroprotectant therapy for cats with renal or GI disease lacking GUE or esophagitis unwarranted, which comprised the majority of cases in this study.

Even among the 32% of cases for which gastroprotectant therapy was deemed appropriate, the medication, dose or frequency prescribed was suboptimal for acid suppression in almost half of the cases. PPIs, such as omeprazole, are superior acid suppressants to H2RAs and sucralfate^51^ and should be administered at 1 mg/kg q12h to maintain gastric pH at the levels and durations needed for esophageal and gastroduodenal healing.^25,52^ However, in this study, an H2RA or sucralfate was often selected over a PPI or a PPI was administered q24h instead of q12h. Omeprazole was often prescribed q24h when dispensed upon discharge, which could reflect the challenges of owners administering oral medications q12h to cats, lack of awareness by the clinician or, less likely, a desire by the clinician to taper the medication accordingly. None of the discharge instructions specifically advised clients to taper the omeprazole, which makes the latter explanation less likely, and could have resulted in abrupt discontinuation and rebound acid hypersecretion in cats receiving omeprazole chronically.^24^ Because H2RAs provide inferior acid suppression relative to PPIs^51^ and rebound hyperacidity has not been observed in cats after the discontinuation of famotidine,^53^ tapering instructions for H2RAs were not examined.

Pantoprazole or omeprazole was the only intravenously or orally administered PPI, respectively, used in this study. This was likely because although other options for PPI medications (esomeprazole, lansoprazole and dexlansoprazole) exist, only one study has studied the efficacy of these alternatives in cats. The results showed that oral esomeprazole was superior to lansoprazole and dexlansoprazole, but optimal acid suppression was only achieved after 4 consecutive days of administering 1 mg/kg q12h. Pantoprazole and omeprazole were also prescribed more heavily, likely because publications have shown superior acid suppression of omeprazole compared with H2RAs in healthy cats.^51,52^ Prescriber preference and clinical experience might also have played a role in the selection of medication, but this was difficult to ascertain retrospectively from medical records. Both PPI and H2RA medications were equally available and accessible to prescribing clinicians and therefore would not have impacted decisions in prescription.

Pantoprazole and omeprazole were typically prescribed at appropriate doses, but the median dose of famotidine was below therapeutic recommendations at 0.7 mg/kg. Clinicians might have the dose reduced to account for the decreased renal excretion in cats with kidney disease;^25,54^ however, this justification was not cited in the medical records. Although the dose of PPI was typically appropriate, only 50% of discharge instructions advised owners on the timing of omeprazole administration relative to a meal. Because the efficacy of a PPI depends on the activity of the gastric parietal cell acid transporters, PPIs should be administered 30–45 mins before meals.^25^ Not instructing pet owners to administer omeprazole as such could have compromised the efficacy of the medication. Similarly, discharge instructions for cats discharged with sucralfate included information on administration relative to meals and other medications in only 12/18 (66.7%) and 3/18 (16.7%) cases, respectively, which could have compromised the mucosal adherence of sucralfate and bioavailability of medications, such as ciprofloxacin, theophylline and doxycycline.

The recommendations on the duration of administration were also lacking in many discharge instructions. In 21% and 44% of cats discharged with omeprazole or famotidine, respectively, administration was recommended indefinitely or until otherwise directed. Continued administration of famotidine can result in a diminished effect on gastric pH secondary to tolerance,^53^ increased costs and unnecessary adverse effects^19^ unbeknownst to pet owners, considering that discharge instructions rarely informed clients of potential adverse effects in this study. When medications are dispensed at this institution’s pharmacy, monographs that include basic medication information and potential adverse effects are supplied to pet owners. However, because clients might not read this material, verbal and written discharge instructions are also recommended to optimize medication adherence and safety.^55,56^

The present study has some limitations. First, the data were retrospectively collected from a single tertiary referral institution, which might not reflect gastroprotectant use at other veterinary hospitals; however, because there were numerous prescribing clinicians, oversampling bias from only one or two prescribing clinicians was avoided. In addition, because many clinicians and students were involved in cases, the completeness and detail of medical records varied, which made it more challenging to infer the rationale behind the prescription in some cases. Furthermore, instructions regarding administration, adverse effects, discontinuation or tapering could have been communicated verbally via telephone or in person and not captured in the medical records. Client compliance also could not be assessed and, therefore, administration of gastroprotectant medications at home was unknown. In addition, the ACVIM consensus statement does not provide an exhaustive list of criteria or diagnoses that warrant the use of gastroprotectants. As such, two board-certified internists with emphases in gastroenterology reviewed the medical records, discussed case scenarios and reached a consensus on the criteria that justified gastroprotectant therapy. Although this approach was subject to biases from the two internists, decisions were led by published evidence, but also clinical experience, which incorporated a realistic clinical practice perspective. Finally, the ACVIM consensus statement was published in October 2018 near the end of the study period. Thus, the misuse of gastroprotectants presented in this study might have improved in the years after its publication.

## Conclusions

The results of the present study revealed the frequent injudicious prescription and administration of gastroprotectants to cats that were presented to a veterinary tertiary referral hospital, and discharge instructions that lacked information about timing and duration of administration, tapering and adverse effects. These findings highlight the need to bring awareness and increase the guidance of veterinarians to the indications for and potential risks of gastroprotectant therapy in cats. These efforts should help minimize the unnecessary use of gastroprotectants with a commensurate decrease in adverse events, accumulated costs and the burden on pet owners to administer gastroprotectant medications to their pets.

## Supplemental Material

Table 1 Grouping of cats according to appropriateness of gastroprotectant medication prescription and medication prescribed
