# Supplementary material for: Evaluation of gastroprotectant administration in hospitalized cats in a tertiary referral hospital
Source: J Feline Med Surg. 2023 Oct 24;25(10):1098612X231201769. doi: 10.1177/1098612X231201769 (PMC10812023; doi:10.1177/1098612X231201769)
Supplement: Table 1 [file sj-docx-1-jfm-10.1177_1098612X231201769.docx]

**Supplementary Table 1: Grouping of cats according to appropriateness of gastroprotectant medication prescription and medication prescribed**

| **Appropriateness of gastroprotectant prescription** | **Count (%)** | **Gastroprotectant prescribed** | **Route of administration of PPI or H2RA** |
| --- | --- | --- | --- |
| **Group 1a (Appropriate)** | 19/110 (17%) | PPI – 18/19 (95%)  H2RA – 0/19 (0%)  Sucralfate – 7/19 (37%) | PPI – IV (11), PO (5), IV and PO (2) |
| **Group 1b (Suboptimal)** | 16/110 (15%) | PPI – 10/16 (63%)  H2RA – 4/16 (25%)  Sucralfate – 8/16 (50%) | PPI – IV (3), PO (5), IV and PO (2)  H2RA – IV (1), PO (3) |
| **Group 2 (Equivocal)** | 7/110 (6.4%) | PPI – 6/7 (86%)  H2RA – 1/7 (14%)  Sucralfate – 0/7 (0%) | PPI – IV (5), PO (1)  H2RA – PO (1) |
| **Group 3 (Inappropriate)** | 67/110 (61%) | PPI – 60/67 (90%)  H2RA – 6/67 (9%)  Sucralfate – 3/67 (4.5%) | PPI – IV (46), PO (7), IV and PO (7)  H2RA – PO (6) |
| **Group 4 (Insufficient Information)** | 1/110 (1%) | PPI – 1/1 (100%) | PPI – IV (1) |

PPI – Proton pump inhibitor

H2RA – Histamine-2 receptor antagonist

PO – Per os

IV - Intravenous
